# Association of chronic kidney disease and risk of hearing loss: a systematic review and meta-analysis

**DOI:** 10.1016/j.bjorl.2026.101861

**Published:** 2026-07-23

**Authors:** Yang Li, Yuanyuan Zhu, Mian Zhang, Ni Li, Delan Zeng, Jingxuan Wei

**Affiliations:** a Guangxi University of Chinese Medicine, Nanning, Guangxi Zhuang Autonomous Region, China; b The First Affiliated Hospital of Guangxi University of Chinese Medicine, Nanning, Guangxi Zhuang Autonomous Region, China

**Keywords:** Chronic kidney disease, Hearing loss, Systematic review and meta-analysis

## Abstract

**Objectives:**

The association between Chronic Kidney Disease (CKD) and Hearing Loss (HL) is intricate. This systematic review and meta-analysis aims to explore the potential association between CKD and the risk of hearing loss.

**Methods:**

We conducted a comprehensive search from the inception of the databases up to February 2, 2025, aimed at exploring the relationship between Chronic Kidney Disease (CKD) and Hearing Loss (HL). Synthesis and meta-analysis of odds ratios, along with their corresponding 95% Confidence Intervals, were performed using Stata 14.0. Funnel plot analysis and Egger’s regression test were applied to assess potential publication bias.

**Results:**

Ten articles involving 1,392,700 participants were included. CKD patients exhibited an increased risk of developing Hearing Loss (HL) (OR = 1.50; 95% CI: 1.13–1.98). Subgroup analysis revealed a higher risk of HL in cohort studies (OR = 1.77; 95% CI: 1.07–2.94) and a greater risk of HL in Asian countries (OR = 1.56; 95% CI: 1.06–2.29), with China showing a higher risk of HL than South Korea (OR = 1.85; 95% CI: 0.93–3.36; *I^2^* = 98.0%). Male CKD patients were more likely to develop HL than females (OR = 1.52; 95% CI: 0.98–2.37). CKD patients aged 45–64 had the highest risk of hearing loss (OR = 1.68; 95% CI: 1.07–2.63).

**Conclusions:**

Our meta-analysis shows that CKD increases the risk of developing HL. These findings provide evidence that CKD should be considered an independent risk factor for HL.

## Highlights

• This study addresses gaps in previous meta-analyses, showing CKD increases HL risk.
• CKD patients in Asia, especially China, have a higher risk of developing HL.
• Male CKD patients were more likely to develop HL than females.
• CKD patients aged between 45 and 64 have a higher risk of developing HL.

## Introduction

Chronic Kidney Disease (CKD) refers to kidney damage or a decline in the estimated Glomerular Filtration Rate (eGFR) to below 60 mL/min/1.73 m^2^, persisting for 3-months or longer.^1^ As a non-communicable disease, CKD has caused a significant number of deaths globally, becoming a major public health issue.^2^ Due to global population growth and aging, the prevalence and incidence of CKD continue to rise, leading to 1.2 million deaths and 28 million years of life lost each year.^3^, ^4^, ^5^ In 2023, the global median prevalence of CKD was 9.5%, with a median mortality rate of 2.4%.^6^ It is estimated that by 2040, CKD will become the fifth leading cause of death worldwide, marking the largest expected increase among all major causes of death.^7^ According to statistics, over 1.5 billion people worldwide suffer from Hearing Loss (HL), making it the third leading cause of disability globally.^8^ Without intervention, it is projected that by 2050, the number of people affected by HL will reach 2.5 billion.^9^ Recent studies have shown that HL can have irreversible effects on children's language development, leading to learning difficulties in school-aged children and employment challenges in adulthood.^10^^,^^11^ In adults and the elderly, HL often leads to social and familial withdrawal, which can further contribute to social dysfunction, limited social engagement, and psychophysiological issues such as cognitive decline and dementia.^12^ The most common otolaryngological complication of chronic kidney disease is damage to the auditory-vestibular system.^13^ Research has shown that the kidneys and auditory structures share a common morphological development origin, suggesting that genetic abnormalities leading to familial hearing loss may also be implicated in kidney disease.^14^^,^^15^ At the cellular and molecular levels, both systems rely on the activity of cilia at their apical surfaces, and renal tubular cells exhibit ion transport mechanisms similar to those of inner ear sensory epithelial cells. Furthermore, they share the same basement membrane network.^15^ Given this close developmental and physiological connection, it is highly probable that chronic kidney disease is associated with an increased risk of hearing loss.

Despite an expanding body of evidence indicating a relationship between CKD and HL, the results across studies have been inconsistent, and a comprehensive quantitative assessment of the strength of this association is lacking. Importantly, to date, there have been no systematic reviews or meta-analyses addressing this issue. To fill this gap in the literature, we conducted a comprehensive systematic review and meta-analysis aimed at exploring the potential association between CKD and the risk of hearing loss.

## Methods

### Protocol and registration

This study was conducted in accordance with the Preferred Reporting Items for Systematic Reviews and Meta-Analyses (PRISMA) guidelines. We performed a systematic review based on a pre-registered protocol (PROSPERO No. CRD42025641289) to ensure the originality of our selected topic.

### Data sources and searches

We conducted searches in several databases (PubMed, Embase, Cochrane Library) from their inception until 9 February 2025. Our search terms for “Chronic Kidney Disease” included “Renal Insufficiency, Chronic [Mesh]”, “Chronic Renal Insufficien*”, “Chronic Kidney Insufficien*”, “Chronic Kidney Disease*”, and “Chronic Renal Disease*”, while those for “hearing loss” included “Hearing Loss [Mesh]”, “Hearing Loss*”, “Hypoacus*”, “Hearing Impairment*”, and “Transitory Deafness*”. The detailed procedures and steps involved in literature retrieval are provided in Supplementary Tables S1–S1.

### Eligibility criteria

Inclusion criteria: (1) Chronic Kidney Disease (CKD) patients were diagnosed based on an eGFR <60 mL/min/1.73 m^2^, insurance records, and hospital diagnostic codes. (2) Patients at risk for HL outcomes were diagnosed using pure-tone audiometry thresholds, insurance records, and hospital diagnostic codes. (3) Study design: Observational studies, which could include case-control studies, cohort studies, or cross-sectional studies.

Exclusion criteria included: CKD patients undergoing hemodialysis treatment, as well as literature with no access to full text or missing raw data, studies with illogical study design protocols, literature that did not report an ethical review process, and meeting abstracts, reviews, and letters, were also excluded.

### Study selection

Two researchers performed the initial screening by reviewing study titles and abstracts, followed by downloading and evaluating the full texts of potentially eligible studies to determine final inclusion based on the eligibility criteria. In the event of any disagreement between the two researchers, a consensus was reached through discussion and consultation with a third researcher.

### Data extraction

Two researchers independently gathered information using a pre-designed checklist. In cases of disagreement, final decisions were made by a third researcher after a thorough review of the full text. The checklist aimed to collect detailed information, including publication details (first author, publication year, country), study characteristics (study type, region, gender, average age/age range, number of cases), exposure and outcome diagnostic criteria, subgroup stratification (including patient age, region, study type, and gender), adjustment for confounding factors, and corresponding 95% Confidence Interval effect sizes (selecting effect sizes adjusted for all available covariates).

### Quality assessment

We assessed the risk of bias using the Newcastle-Ottawa Scale (NOS) for cohort studies, and the Agency for Healthcare Research and Quality (AHRQ) criteria for cross-sectional studies. Studies scoring ≥5 were classified as high quality.

### Statistical analysis

We used Stata 14.0 software for this meta-analysis. Pooled Odds Ratios (ORs) and their corresponding 95% Confidence Intervals (95% CIs) were used as reliable metrics for assessing the risk of depression, regardless of study design. Given the low incidence rates of AR and ADHD in the population, ORs were considered equivalent to Relative Risks (RRs) and Hazard Ratios (HRs) in our analysis.^16^ A fixed-effects model was applied when *I^2^* was ≤50% to combine the effects; otherwise, a random-effects model was used. Sensitivity and subgroup analyses were subsequently conducted to investigate potential sources of heterogeneity. Funnel plots, along with Begg's and Egger's tests, were employed to assess publication bias.^17^

## Results

### Retrieved literature and study characteristics

**Fig. 1:**
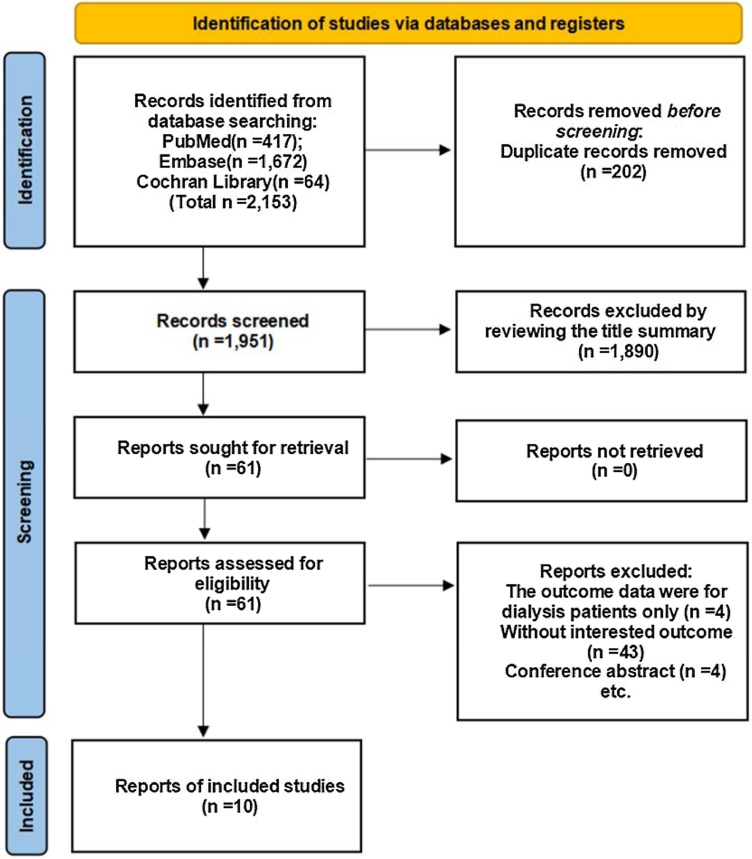
Flow diagram of the search strategy and study selection.

The article selection process is outlined in the PRISMA flowchart shown in Fig. 1. Initially, 2,153 articles were identified through database searches and manual searches. After removing duplicates, 1,951 unique records were critically screened by two independent reviewers based on their titles and abstracts. Following the initial screening, 10 studies^18^, ^19^, ^20^, ^21^, ^22^, ^23^, ^24^, ^25^, ^26^, ^27^ were selected from the pool of records and underwent further full-text assessment. The key characteristics of these studies are summarized in Table 1. Among the eligible studies, 5 were cross-sectional and 6 were cohort studies.

**Table 1:** Basic characteristics of the included studies.

Author	Year	Country	Study type	Sample size	Diagnosis of CKD / HL	Exposures	Outcome	Confounders adjusted	Age
Yihong Zou et al.	2024	United States	Cross-sectional study	Total: 1,204	**Chronic kidney disease:** eGFR <60 mL/min/1.73 m^2^ or ACR ≥30 mg/g.	Chronic kidney disease	Hearing loss	Age, sex, race, education level, marital status, poverty income ratio, BMI, smoking, depression, and cardiovascular disease.	Aged ≥20 years
					**Hearing loss:** Self-reported via NHANES*.				
Kun-Lin Wu et al.	2020	Taiwan, China	Cohort study	Total: 741,720	**Chronic kidney disease**: ICD-9-CM code, 389.1.	Chronic kidney disease	Hearing loss	Age, gender, comorbidities and insured premium.	Aged ≥18 years
					**Hearing loss**: ICD-9-CM code, 389.1.				
Wenwen Liu et al.	2020	China	Cross-sectional study	Total: 12,518	**Chronic kidney disease:** eGFR was used to assess kidney function.	Chronic kidney disease	Hearing loss	Age, gender, education, area of residence, smoking and drinking status, BMI, central obesity, HTN, DM, stroke, high-density lipoprotein cholesterol and low-density lipoprotein cholesterol.	Aged ≥45 years
					**Hearing loss:** Trained investigators asked participants these questions in face-to-face interviews. A participant was considered to have hearing loss if they met one of these criteria: (1) Had a hearing problem, (2) Wore a hearing aid, or (3) Had poor hearing status.				
Ye Ji Shim et al.	2023	Korea	Prospective cohort study	Total: 514,866	**Chronic kidney disease:** Categorized if the participants were diagnosed with CKD (ICD-10 codes: N18) ≥2 times or unspecified kidney failure (ICD-10 codes: N19).	Chronic kidney disease	Hearing loss	Age, sex, physical activity, body mass index, DM, HTN, prevalent cardiovascular disease, alcohol consumption, smoking status, education level and living alone.	Aged ≥40 years
					**Hearing loss:** ICD-10 codes H912 and claim code (E6931-E6937, F6341-F6348).				
Jong-Yeup Kim et al.	2021	Korea	Cohort study	Total: 7,716	**Chronic kidney disease and hearing loss:** Used the NHANES database from 2002 to 2013.	Chronic kidney disease	Hearing loss	Sex, age, residential area, household income, HTN and DM.	Aged <45, 45–64, >64
Sung Keun Park et al.	2020	Korea	Prospective cohort study	Total: 27,731	**Chronic kidney disease:** The estimated glomerular filtration rate (eGFR) was used to assess kidney function.	Chronic kidney disease	Hearing loss	Age, sex, education, income, marital status, DM and HTN.	Aged ≥65 years
Shruti Gupta et al.	2020	United States	Cohort study	Total: 3,921	**Chronic kidney disease:** The glomerular filtration rate was calculated using serum creatinine and cystatin C.	Chronic kidney disease	Hearing loss	Sex, total cholesterol, smoking, waist circumference, education, NSAID* use, loop diuretic use, HTN* and DM*.	Aged 43–84 years
					**Hearing loss:** defined as PTA >25db hearing level.				
Young Joon Seo et al.	2015	Korea	Cross-sectional study	Total: 5,226	**Chronic kidney disease:** diagnosed CKD as an eGFR <60 mL/min/1.73 m^2^	Chronic kidney disease	Hearing loss	Age, sex, smoking, alcohol, BMI, DM, HTN, dyslipidemia and microalbuminuria.	Aged ≥19 years
					**Hearing loss:** Participants were classified as having a hearing loss if the average hearing threshold exceeded 40 dB in each ear.				
Charlene Lin et al.	2013	Taiwan	Retrospective cohort study	Total: 74,842	**Chronic kidney disease:** ICD-9 code 582, 583, 585, 586, and 588.	Chronic kidney disease	Hearing loss	Age, sex, stroke, coronary artery disease, DM, hyperlipidemia, gout.	Aged 0–35, 35–49, 50–64 and ≥65 years
					**Hearing loss:** ICD-10 code H912				
Eswari Vilayur et al.	2010	Australia	Cross-sectional study	Total: 2,956	**Chronic kidney disease:** diagnosed CKD, as an eGFR <60	Chronic kidney disease	Hearing loss	Age, sex, history of noise at work, smoking, education level, history of diagnosed stroke and DM.	Aged ≥50 years
					**Hearing loss:** defined as PTA >25db hearing level.				

eGFR, estimated Glomerular Filtration Rate; ACR, Albumin-to-Creatinine Ratio; PTA, Pure Tone Average; HTN, Hypertension; BMI, Body Mass Index; DM, Diabetes Mellitus; NSAID, Nonsteroidal Anti-Inflammatory Drugs.

### Quality assessment

Cross-sectional studies underwent evaluation based on AHRQ criteria, which included studies explicitly stating the research question, specifying the target study population, and utilizing valid and reliably administered exposure and outcome measures. The results of the quality assessment revealed that no high-risk studies were included after detailed review, as depicted in Supplementary Table S4.

The assessment of all included cohort studies using NOS scores indicated that six studies were classified as high quality, with no low-quality studies included in this meta-analysis (Supplementary Table S5).

### Risk of hearing loss in chronic kidney disease patients

**Fig. 2:**
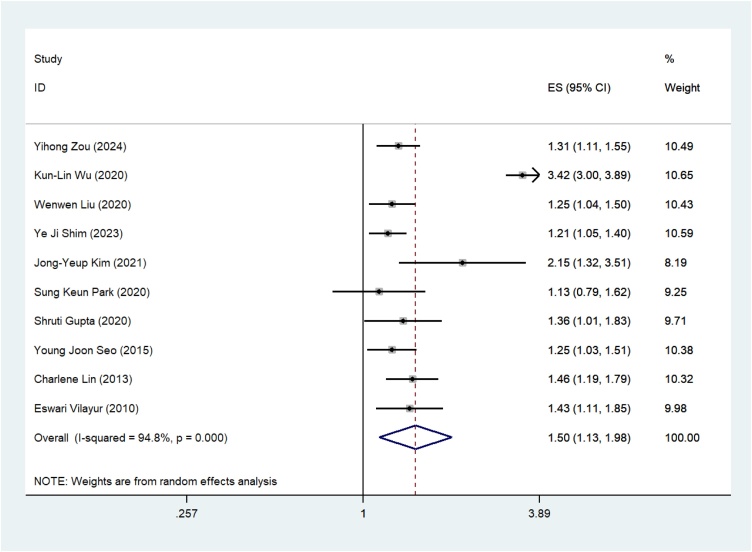
CKD and risk of HL.

A total of 10 studies examined the association between CKD and the risk of developing HL. The results showed that individuals with CKD were more likely to develop HL compared to the general population, with an odds ratio of 1.50 (95% CI: 1.13–1.98; *I^2^* = 94.8%; Fig. 2). Due to the relatively high heterogeneity of the results, we performed a sensitivity analysis (Supplementary Fig. S1) to determine if the heterogeneity was influenced by the exclusion of individual studies. The analysis revealed that excluding any single study did not significantly affect the overall findings (Supplementary Fig. S1). However, the results still exhibited substantial heterogeneity; therefore, we must interpret these findings with caution.

### Subgroup analysis

#### Study type

**Fig. 3:**
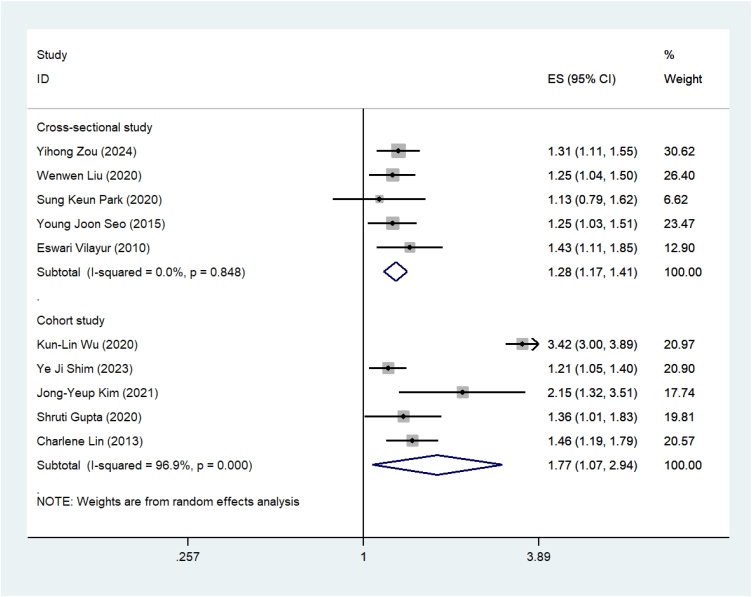
The CKD and the risk of HL depending on the classification by study type.

This analysis included data from 10 eligible studies. We conducted a subgroup analysis based on study design, which revealed that cohort studies had a higher risk of hearing loss in CKD patients compared to cross-sectional studies. Specifically, the risk of hearing loss in the cohort studies was higher (OR = 1.77; 95% CI: 1.07–2.94; *I^2^* = 96.9%) than in the cross-sectional studies (OR = 1.28; 95% CI: 1.17–1.41; *I^2^* = 0.00%; Fig. 3). Sensitivity analysis showed that no single study reversed the pooled effect size, indicating that the results are robust (Supplementary Fig. S2). However, the cohort studies displayed high heterogeneity, so caution is warranted when interpreting these findings.

### Region

**Fig. 4:**
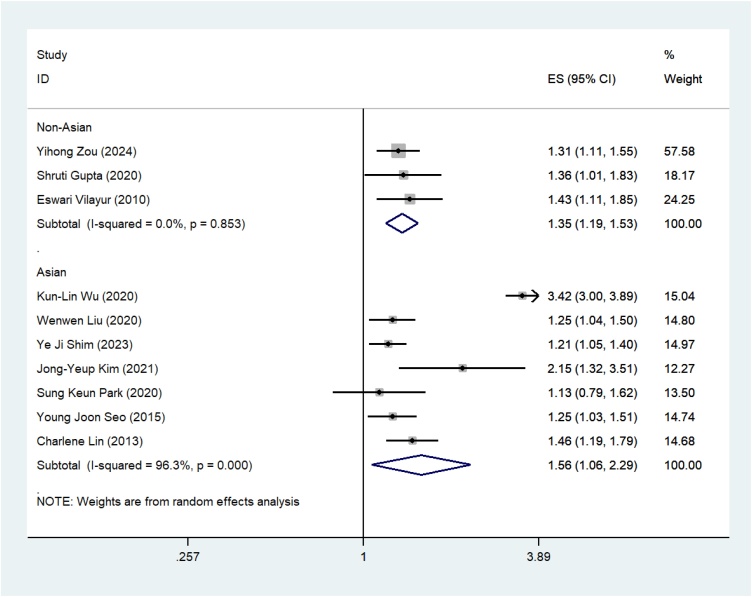
The CKD and the risk of HL depending on the classification by region.

**Fig. 5:**
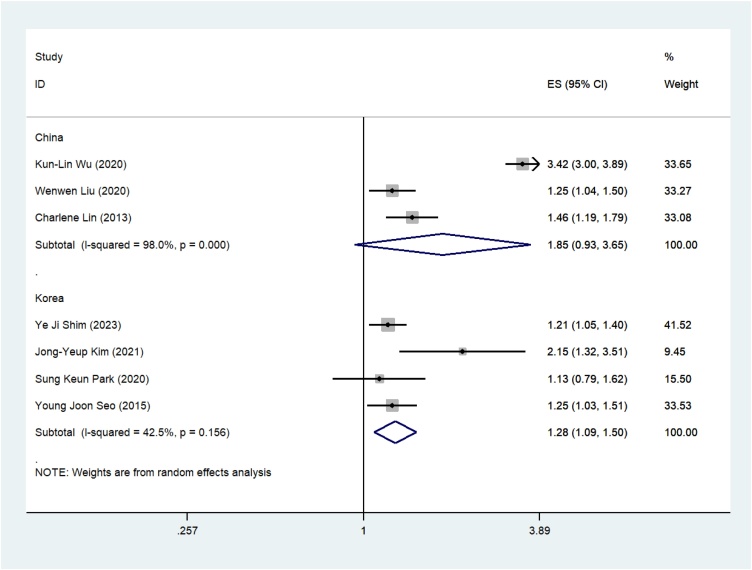
The CKD and the risk of HL depending on the classification by Asian group.

We performed a subgroup analysis based on geographical location, which revealed that CKD patients in Asia had a higher risk of HL compared to those in non-Asian countries. In the Asian group, the risk of HL among CKD patients was OR = 1.56 (95% CI: 1.06–2.29; *I^2^* = 96.3%; Fig. 4), while in the non-Asian group, the OR was 1.35 (95% CI: 1.19–1.53; *I^2^* = 0.0%; Fig. 4). This difference may be attributed to the limited number of studies conducted in non-Asian countries. To further explore the high heterogeneity within the Asian group, we conducted a second level of subgroup analysis, dividing the Asian studies into those from China and South Korea. Specifically, we found that the risk of HL in CKD patients was higher in China (OR = 1.85; 95% CI: 0.93–3.36; *I^2^* = 98.0%; Fig. 5) compared to South Korea (OR = 1.28; 95% CI: 1.09–1.50; *I^2^* = 42.5%; Fig. 5). However, studies from China still exhibited high heterogeneity, so we must exercise caution when interpreting the results from both the Asian and Chinese subgroup analyses.

### Age

**Fig. 6:**
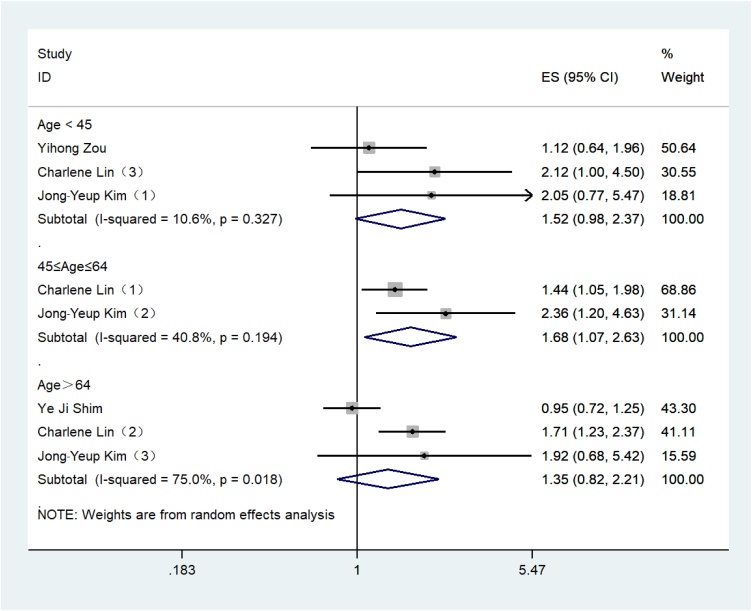
The CKD and the risk of HL depending on the classification by age group.

We conducted a subgroup analysis based on age, ultimately selecting five eligible studies. We divided the participants into three age groups: Age < 45, 45 ≤ Age ≤ 64, and Age > 64. Using a random-effects model to analyze the data from these five studies, we found that CKD patients aged 45–64 had the highest risk of HL (OR = 1.68; 95% CI: 1.07–2.63; *I^2^* = 40.8%; Fig. 5), followed by those under 45-years old (OR = 1.52; 95% CI: 0.98–2.37; *I^2^* = 40.8%; Fig. 6). The lowest risk of HL was observed in CKD patients over 64-years old (OR = 1.35; 95% CI: 0.82–2.31; *I^2^* = 75.0%; Fig. 6).

### Gender

**Fig. 7:**
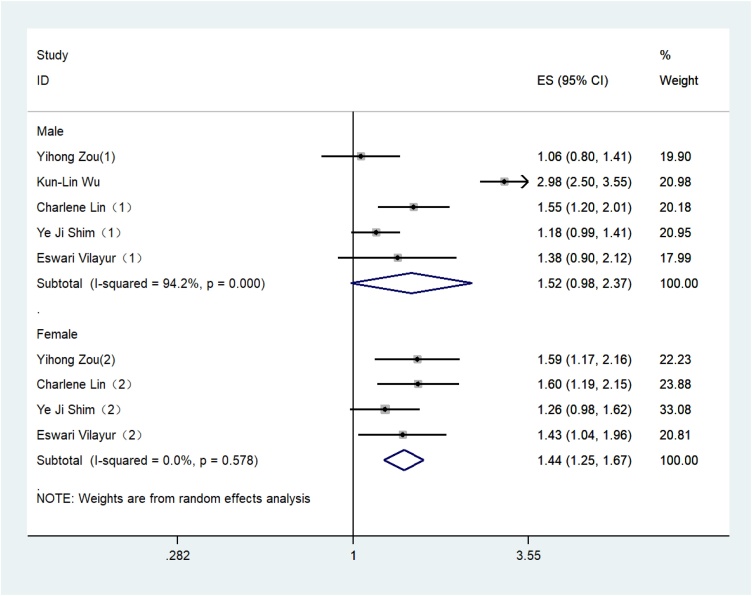
The CKD and the risk of HL depending on the classification by gender group.

We conducted a subgroup analysis based on gender, including data from five studies that examined the risk of HL in CKD patients of different sexes. Specifically, male CKD patients had a higher risk of HL (OR = 1.52; 95% CI: 0.98–2.37; *I^2^* = 94.2%; Fig. 7) compared to female patients (OR = 1.44; 95% CI: 1.25–1.67). However, the male subgroup demonstrated high heterogeneity, so caution is advised when interpreting these results.

### Publication bias

**Fig. 8:**
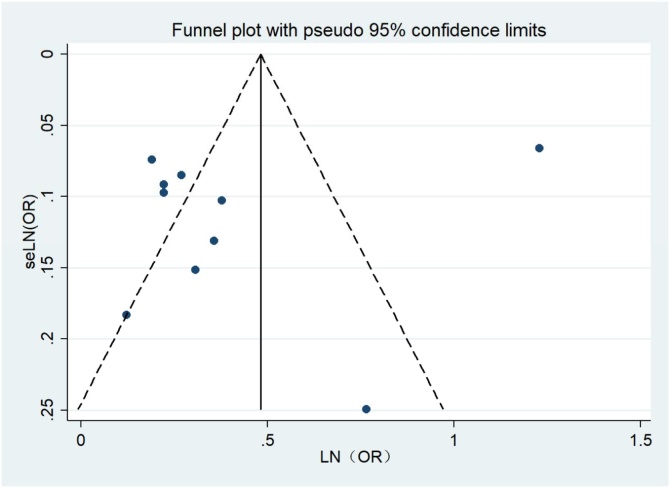
Funnel plot for publication bias.

The funnel plot for CKD was relatively symmetrical, indicating no publication bias. Egger’s test (p = 0.071) revealed no bias for CKD. The funnel plot is shown in Fig. 8.

## Discussion

### Main findings

This meta-analysis included 10 studies involving a total of 1,392,700 individuals, providing a comprehensive evaluation of the association between CKD and HL. We found that the risk of HL was significantly higher in CKD patients compared to those without the condition, suggesting that CKD may be an independent risk factor for hearing decline. Additionally, we observed that the risk of HL was greater in cohort studies than in cross-sectional studies. Among different age groups, CKD patients aged 45–64 had a higher risk of HL compared to those younger than 45 or older than 64. Male CKD patients were at a higher risk of HL than their female counterparts. In terms of geographical differences, CKD patients in Asia had a higher risk of HL than those in non-Asian regions, with Chinese CKD patients in Asia exhibiting a higher risk than their Korean counterparts within the same region.

### Interpretation of findings

A previous review investigated the prevalence of HL in patients with CKD and found that CKD patients are more prone to HL.^13^ However, this article lacked the inclusion of population-based studies and mainly drew conclusions based on mechanisms of disease. In contrast, we have included population-based studies and conducted a more detailed subgroup analysis based on different study types, age, gender, region, and other factors. This approach provides stronger evidence for the association between CKD and HL. Several mechanisms have been proposed to explain the connection between CKD and HL. Some evidence suggests a close developmental relationship between the ear and the kidney, both of which rely on proper tissue patterning during embryogenesis and the normal formation of shared structural components, such as ciliated cells and basement membrane elements. These similarities may help explain the increased risk of hearing impairment in CKD patients. For instance, it is hypothesized that even if genetic mutations responsible for diseases do not disrupt tissue morphogenesis in the ear or kidney, variations in signaling pathways crucial for the development of these shared structural elements may lead to a reduced functional reserve, thereby increasing the risk of HL or CKD later in life.^15^ The cochlear stria vascularis and renal glomeruli are epithelial structures closely associated with the vascular system,^28^ and both express ion channels and transporters involved in the K + cycle, as well as the homeostasis of endolymph K+, Na+, Ca2+, and pH in the inner ear and kidneys.^29^ Early reports have shown that patients with HL often also suffer from chronic renal failure, peritoneal dialysis, and hemodialysis, with concurrent anatomical changes in the labyrinth, such as collapse of the endolymphatic system, edema, and atrophy.^30^ The inner ear is highly sensitive to changes in fluid composition, and electrolyte imbalances caused by chronic kidney disease can disrupt endolymph fluid dynamics, potentially further affecting cochlear function and contributing to HL.^31^ Additionally, some medications used for patients with chronic kidney disease are ototoxic. For instance, aminoglycoside antibiotics, commonly prescribed for treating sepsis and urinary tract infections in chronic kidney disease patients, can cause ototoxicity through damage to the cochlear sensory hair cells and the stria vascularis. This ototoxicity is often considered permanent, although neurodegenerative changes can also occur without damage to the cochlear hair cells.^32^, ^33^, ^34^ Despite the well-known side effects of aminoglycosides, these drugs continue to be widely prescribed and used in many low- and middle-income countries.^35^ This is likely due to their low production costs and affordable prices, combined with lax regulation, making aminoglycoside antibiotics popular in societies with limited purchasing power.^36^ Another commonly used drug in chronic kidney disease patients is furosemide, a loop diuretic, which is employed to treat pulmonary edema and fluid overload. While diuretic-induced ototoxicity is typically regarded as acute and usually resolves after treatment discontinuation, the ototoxic effects of furosemide are often overlooked by nurses and other healthcare providers.^13^^,^^37^ When administered more rapidly or via intravenous push, rapid diuresis-induced ototoxicity is more likely to occur.^13^ When prescribing medications, potential ototoxicity should be carefully considered, especially in patients with pre-existing HL, as it may exacerbate both ototoxicity and clinically relevant risks. The dosage of aminoglycosides should be closely monitored. Alternative antibiotics, such as cefepime, may be considered as substitutes for aminoglycosides, particularly in patients who have previously received multiple courses of aminoglycoside treatment. For patients undergoing aminoglycoside therapy, prophylactic treatment with N-acetylcysteine may be considered, as recent evidence suggests that it can reduce the risk of ototoxicity.^38^

In the subgroup analysis, males with chronic kidney disease were found to have a higher risk of hearing loss compared to female patients. However, there is significant heterogeneity within the male group, and the confidence interval is broad, crossing the null value. Therefore, caution is warranted when interpreting this result. While there is no current basic research indicating that gender differences in chronic kidney disease influence the risk of HL, some studies have found that the atherogenic index of plasma may contribute to the onset of HL.^39^ Furthermore, research has shown that the atherogenic index of plasma can exacerbate chronic kidney disease in male patients.^40^ This could be related to differences in lipid levels between males and females, with the underlying cause potentially linked to the influence of sex hormones on lipid metabolism.^41^, ^42^, ^43^ This may help explain why male patients with chronic kidney disease are at a higher risk of HL compared to females. In addition, subgroup analysis revealed that the risk of HL was higher in CKD patients aged 45–60 compared to those under 45, although the reasons for this difference remain unclear. However, existing surveys have shown that the incidence of CKD sharply increases with age.^44^ Studies have also found that elderly CKD patients tend to have a higher burden of cardiovascular comorbidities and are often in more advanced stages of CKD than younger patients, which increases their risk of adverse outcomes.^45^ Nonetheless, since we only included four studies, caution is needed when interpreting these results. Finally, in the regional subgroup analysis, we found that patients with Chronic Kidney Disease (CKD) in Asia have a higher risk of hearing loss compared to those in non-Asian regions. However, given that a large proportion of the studies we included came from Asia, this may limit the generalizability of our findings to other populations. Therefore, caution is warranted when interpreting the pooled odds ratio for this subgroup, as it may reflect strong epidemiological evidence of an association specific to the Asian region. This observation aligns with previous research, which has similarly reported higher prevalence and dialysis rates for chronic kidney disease in Asia.^46^ These findings highlight the importance of future studies focusing on a more diverse range of ethnic and regional populations to further investigate the relationship between chronic kidney disease and hearing loss across different demographic groups.

### Implications and limitations

Our meta-analysis summarizes the existing evidence regarding the association between Chronic Kidney Disease (CKD) and the risk of Hearing Loss (HL), indicating that CKD may be an independent risk factor for hearing loss. This underscores the need for increased attention to the hearing health of CKD patients, as well as the cautious use of potentially ototoxic medications to prevent irreversible HL. When treating patients with both CKD and HL, it is important to consider the connection between the two. The strong mechanistic and epidemiological links between CKD and HL suggest that infants and children with hearing impairments or organ dysfunction should also be evaluated for kidney abnormalities and dysfunction, and vice versa. Additionally, it seems reasonable to consider hearing assessments for adults with advanced CKD or kidney failure. However, this study has certain limitations. First, the majority of the studies included in our analysis were conducted in Asia, specifically in China and South Korea, which may limit the generalizability of our findings. It is also important to note that the restriction to English-language publications may introduce language bias, potentially omitting relevant studies published in other languages. Furthermore, the exclusion of studies involving hemodialysis patients restricts the generalizability of our findings to this specific subpopulation, which may possess distinct clinical characteristics or outcomes. Additionally, while we observed that male patients have a higher risk of hearing loss compared to female patients, the high heterogeneity, along with a broad confidence interval that crosses the null value and a limited sample size, necessitates caution in interpretation. Nonetheless, this finding may serve as a preliminary indication, suggesting that future research could refine gender subgroup analysis to further explore the risk of hearing loss in chronic kidney disease patients and the underlying mechanisms involved. Moreover, our included studies did not provide detailed information regarding the use of aminoglycoside ototoxicity, and ototoxic medications could be a potential confounding factor. The use of ototoxic drugs among chronic kidney disease patients may further increase the risk of hearing loss. Therefore, future researchers should emphasize the reporting of ototoxic medication usage and strive to avoid the use of such drugs in treatment whenever possible. Although we conducted extensive sensitivity and subgroup analyses, and all included studies accounted for confounding factors, some unexplained heterogeneity remains. This may be attributed to differences in study design and population selection. Future researchers should conduct large-scale, population-based studies in Western populations, as well as in Japanese, Mongolian, and North Korean populations, to further explore the association between chronic kidney disease and HL.

## Conclusions

This meta-analysis indicates that Chronic Kidney Disease (CKD) increases the risk of HL in patients. However, further research is needed to elucidate the underlying pathophysiological mechanisms behind this phenomenon. It is crucial for healthcare providers to be aware of this potential risk when caring for and treating patients. Such awareness could inform the prevention and management of these conditions, reduce their burden on public health, and mitigate their detrimental impact on patients' quality of life.

## ORCID ID

Yang Li: 0009-0000-0040-3465

Yuanyuan Zhu: 0009-0000-1606-7232

Mian Zhang: 0009-0000-7504-9827

Ni Li: 0009-0001-0032-238X

Delan Zeng: 0009-0007-2161-9653

Jingxuan Wei: 0009-0000-6212-6571

## Authors' contributions

All authors contributed to the development of the manuscript. Yang Li and Yuanyuan Zhu designed the study. Jingxuan Wei and Ni Li conducted literature searching with the help of Delan Zeng. Ni Li and Yang Li screened and reviewed the articles. Yang Li assessed the quality of included studies. Mian Zhang and Yang Li extracted the data from included studies. Yang Li drafted the manuscript. Delan Zeng provided guidance and approved the final draft. All authors contributed to the article and approved the submitted version.

## Funding

There is no funding support available.

## Data availability statement

The authors declare that all data are available in repository.

## Declaration of competing interest

The authors declare no conflicts of interest.
