# Supplementary material for: Association of chronic kidney disease and risk of hearing loss: a systematic review and meta-analysis
Source: Braz J Otorhinolaryngol. 2026 Jul 23;92(5):101861. doi: 10.1016/j.bjorl.2026.101861 (PMC13425821; doi:10.1016/j.bjorl.2026.101861)
Supplement: Supplementary file 1 [file mmc1.docx]

**BJORL-D-25-00254_Supplementary Material**

**Table 1 PubMed.**

| No. | Content | Result |
| --- | --- | --- |
| #1 | Hearing Loss [MeSH] Sort by: Most Recent | 81,812 |
| #2 | (((Hearing Loss*[Title/Abstract]) OR (Hypoacus*[Title/Abstract])) OR (Hearing Impairment*[Title/Abstract])) OR (Transitory Deafness*[Title/Abstract]) | 71,786 |
| #3 | (Hearing Loss [MeSH]) OR (((Hearing Loss* [Title/Abstract]) OR (Nasal Catarrh*[Title/Abstract])) OR (Rhinitis[Title/Abstract])) Sort by: Most Recent | 52,298 |
| #4 | (Hearing Loss[MeSH]) OR ((((Hearing Loss*[Title/Abstract]) OR (Hypoacus*[Title/Abstract])) OR (Hearing Impairment*[Title/Abstract])) OR (Transitory Deafness*[Title/Abstract])) | 112,864 |
| #5 | Renal Insufficiency, Chronic[MeSH] | 142,797 |
| #6 | (((Chronic Renal Insufficienc*[Title/Abstract]) OR (Chronic Kidney Insufficienc*[Title/Abstract])) OR (Chronic Kidney Disease*[Title/Abstract])) OR (Chronic Renal Disease*[Title/Abstract]) | 95,741 |
| #7 | (Renal Insufficiency, Chronic[MeSH]) OR ((((Chronic Renal Insufficienc*[Title/Abstract]) OR (Chronic Kidney Insufficienc*[Title/Abstract])) OR (Chronic Kidney Disease*[Title/Abstract])) OR (Chronic Renal Disease*[Title/Abstract])) | 190,645 |
| #8 | ((Hearing Loss[MeSH]) OR ((((Hearing Loss*[Title/Abstract]) OR (Hypoacus*[Title/Abstract])) OR (Hearing Impairment*[Title/Abstract])) OR (Transitory Deafness*[Title/Abstract]))) AND ((Renal Insufficiency, Chronic[MeSH]) OR ((((Chronic Renal Insufficienc*[Title/Abstract]) OR (Chronic Kidney Insufficienc*[Title/Abstract])) OR (Chronic Kidney Disease*[Title/Abstract])) OR (Chronic Renal Disease*[Title/Abstract]))) | 417 |

**Table 2 Embase.**

| No. | Content | Result |
| --- | --- | --- |
| #1 | 'hearing impairment'/exp | 155,261 |
| #2 | 'hearing loss*':ab,ti OR hypoacus*:ab,ti OR 'hearing impairment*':ab,ti OR 'transitory deafness*':ab,ti | 86,216 |
| #3 | #1 OR #2 | 172,018 |
| #4 | 'chronic kidney failure'/exp | 237,133 |
| #5 | 'chronic renal insufficienc*':ab,ti OR 'chronic kidney insufficienc*':ab,ti OR 'chronic kidney disease*':ab,ti OR 'chronic renal disease*':ab,ti | 148,511 |
| #6 | #4 OR #5 | 263,836 |
| #7 | #3 AND #6 | 1,672 |

**Table 3 Cochran Library.**

| No. | Content | Result |
| --- | --- | --- |
| #1 | MeSH descriptor: [Hearing Loss] explode all trees | 1,962 |
| #2 | (Hearing Loss*):ti,ab,kw OR (Hypoacus*):ti,ab,kw OR (Hearing Impairment*):ti,ab,kw OR (Transitory Deafness*):ti,ab,kw | 5,597 |
| #3 | #1 OR #2 | 5,778 |
| #4 | MeSH descriptor: [Renal Insufficiency, Chronic] explode all trees | 9,986 |
| #5 | (Chronic Renal Insufficienc*):ti,ab,kw OR (Chronic Kidney Insufficienc*):ti,ab,kw OR (Chronic Kidney Disease*):ti,ab,kw OR (Chronic Renal Disease*):ti,ab,kw | 22,042 |
| #6 | #4 OR #5 | 24,350 |
| #7 | #3 AND #6 | 64 |

**Table 4 Details of the AHRQ.**

| Study | Year | AHRQ Scores |
| --- | --- | --- |
| Yihong Zou | 2024 | 10 |
| Wenwen Liu | 2020 | 7 |
| Sung Keun Park | 2020 | 9 |
| Young Joon Seo | 2015 | 8 |
| Eswari Vilayur | 2022 | 7 |

**Table 5 The quality assessment of cohort studies.**

| Study | Year | Selection | | Comparability | Outcome | Total |
| --- | --- | --- | --- | --- | --- | --- |
| Cohort studies |  | | | | | |
| Kun-Lin Wu | 2020 | | **** | ** | *** | 9 |
| Ye Ji Shim | 2023 | | **** | ** | *** | 9 |
| Jong‑Yeup Kim | 2021 | | **** | ** | *** | 9 |
| Shruti Gupta | 2020 | | **** | ** | *** | 9 |
| Charlene Lin | 2013 | | **** | ** | *** | 9 |

The NOS scale was used to evaluate the quality of the cohort studies and case control studies.

**Figure 1 Sensitivity analysis of the risk of hearing loss caused by** **chronic kidney disease.**

**Figure 2 Sensitivity analysis of the risk of hearing loss caused by chronic kidney disease on the classification by study type.**
